# *Aquilaria* Species (Thymelaeaceae) Distribution, Volatile and Non-Volatile Phytochemicals, Pharmacological Uses, Agarwood Grading System, and Induction Methods

**DOI:** 10.3390/molecules26247708

**Published:** 2021-12-20

**Authors:** Yichen Wang, Mubasher Hussain, Zhenbin Jiang, Zhaohong Wang, Jing Gao, Fengxian Ye, Runqian Mao, He Li

**Affiliations:** 1 College of Life Science and Biopharmaceuticals, Guangdong Pharmaceutical University, Guangzhou 510006, China; wyc1311771587@126.com; 2 Guangdong Key Laboratory of Animal Conservation and Resource Utilization, Guangdong Public Laboratory of Wild Animal Conservation and Utilization, Guangdong Engineering Research Center for Mineral Oil Pesticides, Institute of Zoology, Guangdong Academy of Sciences, Guangzhou 510260, China; wangzh@giz.gd.cn (Z.W.); gaoj@giz.gd.cn (J.G.); yefengxian2021@126.com (F.Y.); 3 Guangdong Xialiang Health Regimen Agricultural Technology Co., Ltd., Guangzhou 510445, China; yl455191986@126.com

**Keywords:** agarwood, chemistry, medicinal plant, pharmacological effects, phytochemicals, research advances

## Abstract

Agarwood is a highly valuable fragrant wood of *Aquilaria* spp. (Thymelaeaceae) which has been widely utilized in traditional medicine, religious rites, and cultural activities. This study summarizes a review on the identification of *Aquilaria* cultivars, volatile and non-volatile phytochemicals, pharmacological uses, and agarwood grading system to determine its quality, and different agarwood induction methods. Due to the highly demanding and depleted natural resources, the research on agarwood is still insufficient, and it has broad research and development prospects in many industries. However, due to the significant scientific nature of agarwood application, developing high-quality products and drugs from agarwood have become highly important, while no one has discussed in detail the phytochemicals uses and provided a summary until now. The main phytochemicals of agarwood include terpenoids, dominated by sesquiterpenes. For centuries, terpenoids have been used in traditional Chinese medicine and have been shown to possess various pharmacological properties, including bacteriostatic, antibacterial, sedation, analgesia, anti-inflammation, anti-asthmatic, hypoglycemic, antidepressant, and many others. Alongside biological activity screening, phytochemical advances and pharmacological research have also made certain progress. Therefore, this review discusses the research progress of agarwood in recent years and provides a reference basis for further study of *Aquilaria* plants and agarwood.

## 1. Introduction

**Figure 1:**
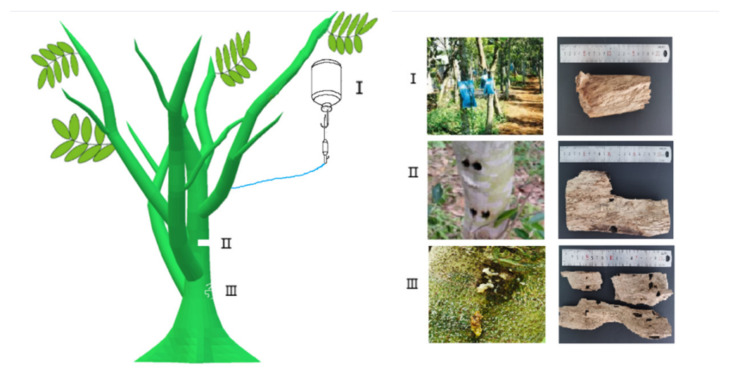
Wound tissues and agarwood sample collection from *Aquilaria sinensis*.

*Aquilaria sinensis* (Lour.) Gilg is the resin-containing wood of the *Aquilaria*. Agarwood is a traditional Chinese medicine included in the 2020 edition of Chinese Pharmacopoeia [1]. There are about 17 species of agarwood in the world, most of which are distributed in China, India, and Southeast Asian countries. In recent years, the majority of agarwood, headed by *Aquilaria sinensis* (Lour.) Spreng, has continuously attracted attention. Agarwood plants are likely to secrete a variety of secondary metabolites when they are damaged, including by lightning, chopping, burning, moth-eating, or microbial invasion of natural factors [2]. The traditional artificial agarwood formation techniques are based on physical methods such as cutting and digging holes. Modern artificial agarwood formation techniques are mainly biochemical methods, such as chemical reagent invasion and bacteria inoculation, which are deposited in the xylem after a complex agarwood formation process after wound formation [3,4]. There are few reports about artificial agarwood, mostly focused on drilling and chemical reagent dripping, but insect infestation is the best method for producing natural agarwood (Figure 1). In recent years, there are many kinds of agarwood commodities and crafts in the market, and the phenomenon of counterfeit mixing is serious. At present, there are many ITS (Internal Transcribed Spacer) intergenic regions for the identification of agarwood species, and the research on agarwood has made some progress, but its identification method is not yet mature. At present, a variety of methods have been established to control the quality of medicinal materials, including the coloration of chemical reagents, the content of alcohol extracts, the content of agarwood tetraol, the content of chromone, the HPLC fingerprint of alcohol extract, etc. Most of the effective components of agarwood are focused on the study of volatile oil, chromone, and sesquiterpene. Agarwood is a valuable traditional Chinese medicine and natural high-grade spice in the world. It has the effects of relieving pain, asthma, and vomiting. It has significant effects when combined with other traditional Chinese medicines such as Bawei Chenxiang Powder and Chenxiang Tongbian Powder. This study focused on pharmacological and clinical research of antibacterial, bacteriostatic, anti-tumor, antidepressant, and antioxidation, as well as the application for cardiovascular and cerebrovascular diseases [5]. However, there is a lack of further research on the mechanism of pharmacological action. In this paper, a comprehensive review was made on the species and identification, quality control, chemical composition, pharmacological action, compatibility, and artificial agarwood to provide a reference for further research and development. It also provides a scientific basis for the development and application of precious medicinal material. There are 17 *Aquilaria* spp distributed around the world, as shown in Table 1.

Type I: agarwood formed by chemical reagent dripping. Type II: agarwood formed by using a nail to dig a hole in the agarwood. Type III: agarwood induced by insects forming irregular wormholes for agarwood production.

## 2. Economic Value and Aquilaria Species Distribution

### Economic Value

**Figure 2:**
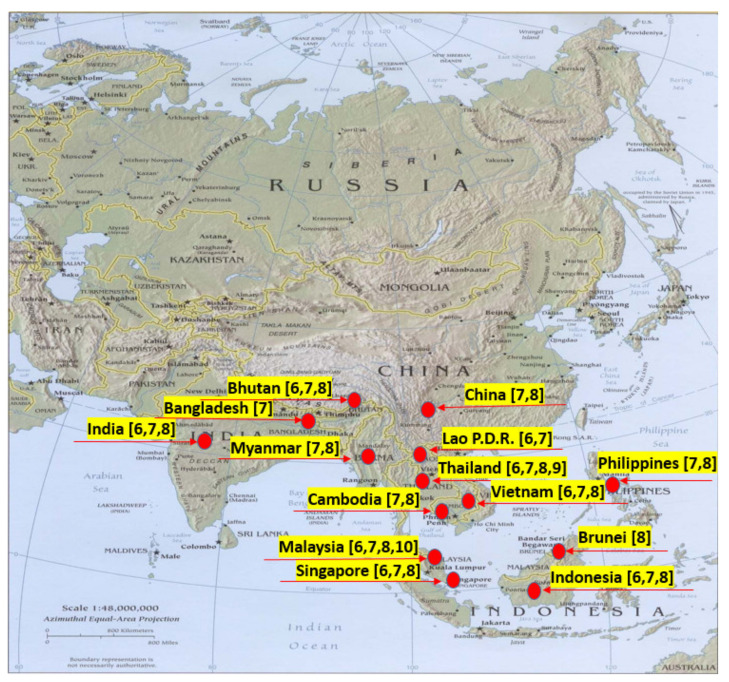
*Aquiliria* spp. Asiatic distribution map. Red spot showing the distribution in different countries.

High-quality agarwood can be sold at US $100,000 per kg, while those of poor quality can only be sold at US $100 per kg. As a high-quality and high-grade agarwood essential oil, it can even be sold for US $1500 per 11.7 g [11,12,13]. The higher the grade of agarwood, the richer the layers of aroma. The best agarwood fragrance is mellow and sweet, full of penetration and persistence, and the powdery waxy material on the surface can be scraped off and kneaded it into a ball. Its aroma is regarded as a symbol of high quality. There are various kinds of volatile components, each with a unique aroma [12,14,15]. *Aquiliria* spp. Asiatic distribution map is shown in Figure 2.

## 3. Aquilaria Identification Methods and Phytochemicals

### 3.1. Identification Methods

There are many kinds of agarwood trees, and their morphological characteristics are very similar, which increases the difficulty in the identification of agarwood. Even if there are different batches of agarwood from the same producing area and different producing areas, the chemical constituents measured by the same experimental method are very different [16]. It is necessary to establish a set of systematic and accurate identification methods. At present, the differences in intragenic regions of Internal Transcribed Spacer (ITS), ITS1, and ITS2 are often used to distinguish different species and genera. For example, Zou et al. [17] used a variety of different extraction methods for identifying different agarwood species by the ITS2 sequence analysis method, using its DNA. The results showed that the DNA barcode sequence based on ITS2 can accurately identify the real and fake species, and the species can be identified by sequence comparison. The similarity of the comparison result with the original agarwood is up to 100%, which proves that this method can effectively distinguish true and fake products and the relationship between species and genera. Meanwhile, Niu et al. [18] identified the sequence-specific regions (ITS1 and ITS2) using PCR amplification. The results showed that there were various sequence-specific regions in the rDNA sequence of agarwood. Shen et al. [19] analyzed the sequence-specific regions (ITS sequence of DNA), and the different regions were distinguished by sequence alignment.

### 3.2. Phytochemicals

The major chemical constituents from *Aquilaria* plants are sesquiterpenoids and chromones (Table 2, Table 3 and Table 4). These are divided into two categories: (A) Volatile compounds of agarwood and (B) Non-volatile compounds of agarwood.

#### 3.2.1. (A) Volatile Compounds of Agarwood

Aromatic group

Aromatic volatile compounds are another important component in agarwood. For example, benzylacetone, which has been widely studied, is regarded as a landmark substance in aromatic components. Chen et al. [20] used the GC-MS and found that chloroform could effectively extract and separate aromatic compounds. Zhang et al. [21] isolated a large number of aromatic compounds from the volatile oil, in which the mass fraction of benzylacetone was the highest, reaching 19.51%. There are great differences in the mass fraction of aromatic compounds in different species. Tajuddin and Yusoff [22] found that the proportion of aromatic compounds in Malaysia agarwood volatile oil is larger than that of other species, of which 4-phenyl-2-butanone accounts for 32.1% of the volatile oil. Mei et al. [23] also isolated some aromatic compounds from agarwood, such as benzylacetone, 2,4-di-tert-butylphenol, 3,5-di-tert-butylphenol, and 4-methoxyphenylacetone (Table 2). These chemical structures were isolated on the basis of spectroscopic evidence with no cytotoxic activity against different cell lines [23].

#### 3.2.2. (B) Non-Volatile Compounds of Agarwood

Fatty acids

Lin et al. [24] used GC-MS to extract the agarwood produced by the nailing method, burrowing method, and cutting method with ether, and it was concluded that the agarwood in the cutting method was mainly fatty acid. Bhardwaj [25] found stearic acid, oleic acid, linoleic acid, and palmitic acid in the agarwood volatile oil by GC-MS. Mei et al. [26] isolated many kinds of fatty acids, such as hexadecenoic acid, tridecanoic acid, octadecenoic acid, and so on.

Chromones

These compounds mainly exist in the *Aquilaria*, and they are volatile constituents of agarwood (Table 3). The dimeric 2-(2-phenylethyl) chromones of agarwood lead to the identification by structure. Liu et al. [27] isolated different kinds of 2-(2-phenylethyl) chromones from agarwood. Mei et al. [28] analyzed the extracts by GC-MS after extracting high-quality agarwood with ether, and found that the relative content of 2-(2-phenylethyl) chromone compounds was 60% in high-quality agarwood. Xia et al. [29] and Yang et al. [30] used a spectrophotometer to evaluate the quality of agarwood and determine the absorbance value of 2-(2-phenylethyl) chromones at the wavelengths of 230 nm and 250 nm. Zhang et al. [31] obtained various derivatives of 2-(2-phenylethyl) chromones by co-fermentation of endophytic strains with agarwood. Thirteen 2-(2-phenylethyl) chromone derivatives were obtained by ethanol extraction, elution, and purification, and then bioassay for antibacterial activity. The results showed that some of 2-(2-phenylethyl) chromone derivatives had good inhibitory effect on *Staphylococcus aureus* [32]. Yang Lin also used a spectrophotometer to isolate different chromones, such as 2-[2-(4-hydroxyphenyl)ethyl]chromone, 5,6,7,8,-tetrahydroxy-5,6,7,8-tetrahydro-2-[2-(4-methoxyphenyl)ethy]-chromone, *Rel-*(1*AR*,2*R*,3*R*,7*bS*)-1a,2,3,7b-tetrahydro-2,3-hidydroxy-5[2-(4-methoxyphenyl)ethy]-7*H*-oxireno[f][1]benzophran-7-one and oxidoagarchromones (Table 3), which showed the mechanism of analgesia and sedation related to the regulation of gene expression of the GABAA receptor, GABAA receptor function, and promotion of Cl^−1^ influx.

Terpenoids

Terpenoids are compounds derived from mevalonic acid that have two or more isoprene units in their basic carbon frame. The major components of agarwood are terpenoids, which include sesquiterpenes and diterpenes. One of the criteria used to evaluate the quality of agarwood is the content of sesquiterpenes and triterpenes (Table 4). Sukkaew et al. [36] isolated and identified volatile compounds from agarwood by GC-MS. Yang et al. [37] isolated and identified a monoterpene derivative [(-)-bornyl ferulate]. According to the chemical structure, sesquiterpenes isolated from agarwood are divided into different categories including agarofurans, eudesmanes, eremophilanes, guaianes, agarospirols, and cadinanes [38,39]. Several kinds of components with a high content (white caryophyllus, white caryophyllenol, and α-white fragrant alkene, etc.) were calculated by the normalization method [40], extracting sesquiterpenes from the “whole-tree agarwood-inducing“ technology in artificial agarwood. Jin et al. [41] have found that the structures of sesquiterpenes are similar, so it is difficult to separate and purify sesquiterpenes by conventional separation methods, such as chromatographic column separation and alcohol extraction. Ismail et al. [42] also found that all the volatile oil chemical structures were mainly composed of sesquiterpenes, such as α-agarofuran, (5*S*,7*S*,10*S*)-(-)selina-3, (+)-(4*S*,5*R*)-dihydrokaranone, α-guaiene, agarospirol, and 8-β-*H*-dihydrogmelofuran (Table 4).

By using silica gel column chromatography to isolate a variety of compounds from traditional Chinese medicinal agarwood, triterpenes (e.g., Ivy sapogenin and 3-oxo-22-hydroxyhopane, etc.) were isolated by recrystallization [43]. Various triterpenoids (approximately 14) were separated and purified from agarwood, including the first discovery of hydroxy-domperidone [44]. Tian and Haigang [45] used petroleum ether and ethanol to extract 10 diterpenoids from agarwood.

Steriods

Some steroids, such as β-sitosterol and 12 flavonoids, were isolated [48].

Flavonoids

Qi et al. [49] isolated 31 flavonoids, including flavonoid glycosides with anti-inflammatory activity. Chen et al. [50] isolated lignans and phenylpropanoids from agarwood, while Hendra et al. [51] and Li et al. [52] isolated several types of phenolic acids including p-methoxyphenylpropionic acid, 4-hydroxyphenylpropionic acid, and others.

Alkaloids

Chen et al. [53] isolated alkaloids from the wood of agarwood. Qi et al. [49] isolated benzophenones from agarwood and found the compound iriflophene, which can inhibit the respiratory burst of neutrophils.

### 3.3. Pharmacological Uses

A variety of phytochemicals from agarwood has obvious pharmacological uses and has always been an interesting topic among researchers and scientists (Table 5). The majority of phytochemicals from *Aquilaria* plants are sesquiterpenoids and chromones, which have been widely used in the pharmacological industry. Some of the pharmacological uses of these compounds are described below.

#### 3.3.1. Antibacterial

Dahham et al. [54] used the sesquiterpene component (β-caryophyllene) against six kinds of human pathogenic bacteria and two kinds of fungi. It was found that β-caryophyllene had an inhibitory effect on the growth rate of bacteria, while the antibacterial activity of Gram-positive bacteria was higher than that of Gram-negative bacteria. The volatile oil extracted by Mei et al. [32] was determined by the agar diffusion method. The results showed that the volatile oil had antibacterial activity, especially against methicillin-resistant *Staphylococcus aureus*. Wang et al. [55] carried out antibacterial experiments with 5-deoxylongiferol isolated from agarwood. The results showed that the compound had an antibacterial effect on *Staphylococcus aureus* and *Ralstonia solanacearum*.

#### 3.3.2. Anti-Tumor

The volatile oil of agarwood contains a variety of anti-tumor components, which have inhibitory effects on many kinds of cancer cells. For example, 2-(2-phenylethyl) chromone compounds were isolated from agarwood for the cytotoxic activity test. It was found that these compounds had inhibitory activity on five kinds of human tumor cell lines [56]. Chen et al. [57] used chloroform to extract the active substances from agarwood and found that the extract had anti-tumor activity against four kinds of cells (IC_50_ = 11.11−58.55 μg mL^−1^). Durham et al. [58] used the essential oil to carry out anti-tumor experiments in nude mice and found that the essential oil had an inhibitory effect on colon cancer cells.

#### 3.3.3. Analgesia, Sedation, Anti-Inflammation

As a breach power medicine, agarwood has the effect of activating breach power and relieving pain. Wang et al. [59] conducted hypnotic experiments with alcohol extract of agarwood and the volatile oil combined with pentobarbital sodium. It was found that both of them could significantly increase the rate of falling asleep and prolong the sleeping time in mice. In addition, it was also found that the volatile oil in agarwood could significantly shorten the falling asleep latency. Wang et al. [60] explored the potential mechanism of essential oil on the γ-aminobutyric acid (GABA) system and found that the essential oil has a sedative and hypnotic effect; its mechanism is related to the regulation of GABAA receptor gene expression, mainly by enhancing the expression function of GABAA receptor and promoting Cl^−1^ influx. Gao [61] studied the anti-inflammatory activity of the extracted essential oil. After repeated intragastric administration, it was found that the essential oil could alleviate the inflammation induced by lipopolysaccharide.

#### 3.3.4. Relieving against Cough and Asthma

Agarwood has some significant effects in relieving cough and asthma. Wu et al. [62] treated cough with agarwood Bawei Powder and found that it cannot only relieve the symptoms of cough, but also treat asthma. Zhou et al. [63] found that alcohol extract could effectively prolong the latent period of a cough caused by concentrated ammonia, and reduce coughing.

#### 3.3.5. Antidepression and Anxiety

Agarwood has an effect in the treatment of anti-depression and anxiety. Wang et al. [64] found that the extracted essential oil from agarwood can effectively inhibit depression and anxiety, indicating that its mechanism may be related to the inhibition of some gene expression. Yang et al. [47] found that diterpenoids isolated from agarwood can effectively inhibit synaptic reuptake of serotonin and norepinephrine, showing obvious antidepressant activity.

#### 3.3.6. Anti-Oxidation and Anti-Aging

Agarwood is a world-famous spice, known as the “king of incense “, and is an important raw material for the agarwood and cosmetics industry. Lin et al. [65] found that agarwood’s alcoholic extract from agarwood leaves had antioxidant and anti-aging activity. Lee et al. [66] found that the alcohol extract has a neuroprotective effect on stress-induced oxidative damage in the hippocampus. Xie et al. [67] found that agarwood volatile oil, at a certain concentration, can significantly reduce the oxidative damage caused by hydrogen peroxide on mouse adrenal pheochromocytoma monoclonal cells, and has a good antioxidant effect.

#### 3.3.7. Effect on the Cardiovascular System

Yang et al. [68] found that Bawei Chenxiang Powder can enhance the hypoxia tolerance of cardiomyocytes, which has a good preventive and therapeutic effect on some common heart diseases. Sugiyama et al. [69] found that several 2-(2-phenylethyl) chromones isolated from incense could inhibit PED 3A in phosphodiesterase (PDEs). Chunyan et al. [70] found that Bawei Chenxiang Powder has a protective effect on the rat model of myocardial ischemia.

#### 3.3.8. Clinical Application

Agarwood is mostly used in the form of a clinical drug, and it has obvious curative effect in the cardio-cerebrovascular system, urinary system, respiratory system, and against other diseases. After continuous treatment of patients with bronchial asthma with Bawei Chenxiang Powder (agarwood, nutmeg, jujube, travertine, frankincense, radix aucklandiae, chebula, kapok), it was found that the total effective rate of patients with Bawei Chenxiang Powder was higher than other medicines, and this condition was better relieved and controlled [71]. Gao [72] described that the patients with angina pectoris (a coronary heart disease) were treated with conventional medicine and Bawei Chenxiang Powder. In the experimental group of 50 patients, the effective rate with Bawei Chenxiang Powder was higher (26.0%) than that of patients treated with routine western medicine, after continuous administration for four weeks. This indicates that Bawei Chenxiang Powder has a significant effect in the treatment of angina pectoris caused by coronary heart disease. Wang el al. [73] treated constipation by grinding Chenxiang Tongbian Powder (agarwood, atractylodes macrocephala, semen raphani, rhubarb, mirabilite, fructus aurantii, peach kernel, radixpaeoniae alba, astragalus membranaceus, magnolia officinalis) and applying it on the navel, combined with acupuncture. It was found that the total effective rate of 30 patients treated with Chenxiang Tongbian Powder was higher (13.33%) than that of patients treated with polyethylene glycol electrolyte orally, and the symptoms of the patients were significantly relieved after two weeks of treatment.

### 3.4. Grading System for Agarwood Identification

The morphological grading system of agarwood is shown in Table 6. At present, the incense sold in the market comes from a wide range of sources, which needs to be processed before it can be used in medicine. It is difficult to distinguish its quality by observing its morphological characteristics, and the handicrafts of incense are even more difficult to identify. It is almost impossible to identify through the naked eye, the circulation in the market is more chaotic, and the phenomenon of adulteration and fraud is more common. The Chinese Pharmacopoeia (2020 edition) indicates that the content of the ethanol extract of resin shall not be less than 10%, and the content of agarotetrol (C_17_H_18_O_6)_ shall not be less than 0.1% [1]. Xie et al. [74] digitized the chemical composition extracted from HPLC-Q-TOF-MS fingerprints, and the quality was evaluated according to the proportion of components. Ismail et al. [75] extracted and separated the volatile oil of Malaysia agarwood. After analysis and screening, the content of γ-eucalyptus oleol was determined as the quality standard of agarwood volatile oil. Chen et al. [57] detected the incense in different fragrance processes by GC-MS, analyzed the different chemical components, distinguished the incense in different ways by the method of grey relational analysis, and, finally, reflected the quality of incense by ranking. The several chemical components of agarwood were isolated by high-performance liquid chromatography (HPLC). After evaluation and analysis, the content of agaropiric acid in incense was selected as the index to establish a standard method for evaluating the quality of artificial incense, with the content of agaropiric acid as the evaluation standard [76].

### 3.5. Agarwood Induction Technique

#### 3.5.1. Natural Induction

With the rapid development of medicine, the demand for incense resources is increasing day by day, and it is even more valuable as medicinal material. Most of the agarwood resources mainly come from the wild, and agarwood needs to be damaged and can be harvested for a long time, and the number of agarwood resources that can be harvested is decreasing day by day.

#### 3.5.2. Artificial Induction

**Figure 3:**
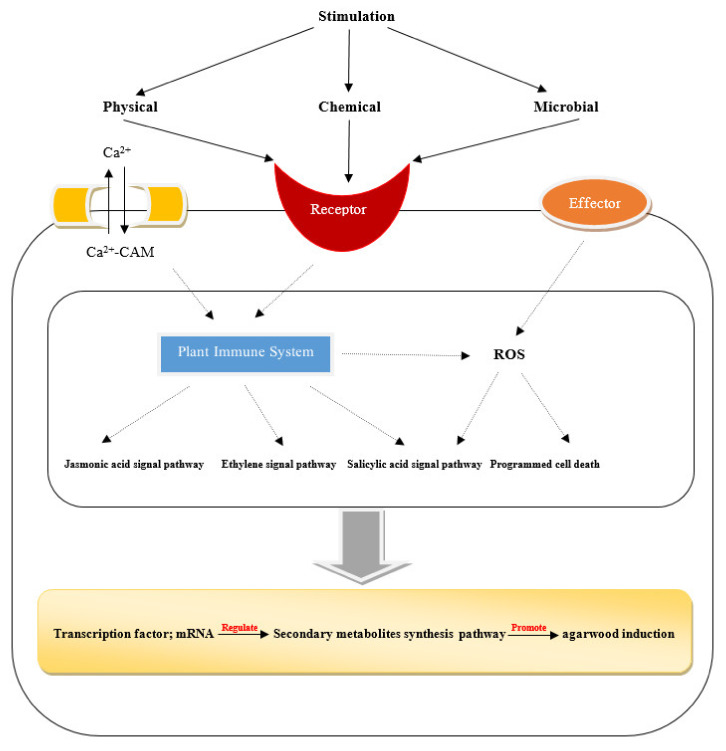
A schematic diagram showing the molecular mechanism of agarwood induction.

The molecular mechanism of agarwood induction is shown in Figure 3. Artificial incense will replace wild incense to become the main source. The artificial incense-forming technology mainly includes the physical injury method, chemical reagent method, biological planting method, and many more [2]. With the development of incense forming technology, most of the artificial incense is mainly composed of chemical techniques. Zhao and Fang [77] evaluated the quality of agarwood produced by the whole-tree agarwood-inducing technology, and preliminarily considered that the quality of incense produced by this technology conformed to the standard of the Chinese Pharmacopoeia. However, the quality of artificial incense is still different from that of natural incense, it is far from the ratio of high-quality insects in natural incense, and there are few reports on how to use insect microorganisms to cause damage to the incense to form high-quality insect incense. The method of using artificial intervention to make the fragrance quality close to that achieved using natural insects is still a blank, so it is necessary to combine incense with insect microorganisms. To produce the incense using high-quality insect leakage is an area that still requires considerable effort. At present, the studies on artificial incense technology are limited, a problem needs to be solved. Therefore, there is a need to establish a standardized incense base and explore the method of combining animals and plants to improve incense technology and provide high-quality medicinal resources.

### 3.6. Future Perspectives and Limitations

There are still many problems to be solved: (1) Because agarwood is a valuable medicinal material, the commodity price is expensive, and the mixing of fake and inferior products in the market increases. It is difficult for people to identify these inferior products with the naked eye. It is even more difficult to identify when processed into handicrafts, and the mixing of adulterated products seriously affects the safety of clinical drug use of agarwood. How to strengthen market supervision and how to establish an accurate and rapid identification of fake and shoddy products is an urgent problem to be solved. (2) Agarwood ranks first among the four famous incenses of agarwood, sandalwood, ambergris, and musk. It has many effects, such as relieving pain, vomiting, asthma, etc., and is widely used in the clinic, such as for anti-bacteria and anti-inflammation, sedation and analgesia, and relieving cough and asthma, to name but a few. However, the specific active substances and pharmacological mechanisms of various pharmacological actions have not been confirmed, which greatly limits the development and utilization of incense resources. Therefore, it is necessary to further study the effective components of agarwood and explore the action mechanism of its active substances. (3) With the massive felling of natural incense, and it will take several years, or even more than ten years, to replace. As the availability of natural incense gradually decreases, artificial incense will become the main source, but the technology of artificial incense is still unknown. Compared with insect incense, there is still a large gap between normal incense and insect incense, and methods to produce high-quality agarwood incense still need to be further explored. At the same time, large-scale planting and scientific cultivation is also an urgent technical problem to be solved.

## 4. Conclusions

Agarwood has great development potential, and its application has a broad prospect. In this article, we talked about the varieties, regional distribution, and current main identification methods of agarwood, discussed some chemical components contained in agarwood, and enumerated the biological pharmacological effects of its components. This report also combined the clinical application of other medicinal materials and classified the advantages and disadvantages of the incense sold in the market. Finally, we provided an example of the agarwood induction mechanism to yield high quality incense.

Currently, the research and development of agarwood are still insufficient, especially in areas such as the rapid identification of mixed and inferior products, the mechanism of pharmacological action, the development of new products, the optimization and development of agarwood-inducing technology, etc. New techniques have been tried, and some progress has been made, in artificial planting and the improvement of agarwood-inducing technology, but ways to improve the scientific utilization value of incense and how to exert its greater value in combination with medicine, agronomy, and insect microbiology still need to be discussed.

## Figures and Tables

**Table 1:** The species of *Aquiliria* and its distribution.

Species	Origin	References
*Aquilaria malaccensis* Lam.	India, Myanmar, Malaysia, Indonesia, Philippines	[6,7,8]
*Aquilaria sinensis* (Lour.) Gilg	China	[7,8]
*Aquilaria microcarpa* Baill	Indonesia	[6,7,8,9]
*Aquilaria apiculata* Merr	Philippines	[8]
*Aquilaria baillonii* Pierre ex Lecomte	Cambodia, Thailand, Laos, Vietnam	[6,8]
*Aquilaria banaensis* P.H.H6	Vietnam	[6,7]
*Aquilaria beccariana* Tiegh	Indonesia	[6,7]
*Aquilaria citrinicarpa* (Elmer) Hallier f.	Philippines	[6,8,10]
*Aquilaria cumingiana* (Decne) Ridl	Malaysia	[8]
*Aquilaria khasiana* Hallier f.	India	[6,7,8]
*Aquilaria apiculata* Merr	Philippines	[8,10]
*Aquilaria parvifolia* (Quisumb) Ding Hon	Philippines	[6,10]
*Aquilaria rostrata* Ridl	Malaysia	[6,7,9]
*Aquilaria rugosa* Kiet Kessler	Vietnam	[8]
*Aquilaria subintegra* Ding Hon	Thailand	[8,9]
*Aquilaria urdanetensis* (Elmer) Hallier f.	Philippines	[8,10]
*Aquilarla yunnanensis* S.C. Huang	China	[7,8]

**Table 2:** Aromatic compounds from agarwood.

Name	Contents (%)	Chemical Structure	Reference
Benzylacetone	0.95	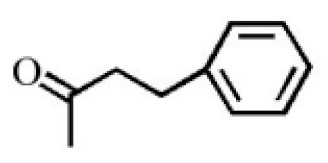	[23]
2,4-Di-tert-butylphenol	4.15	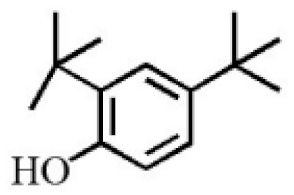	
3,5-Di-tert- butylphenol	2.70	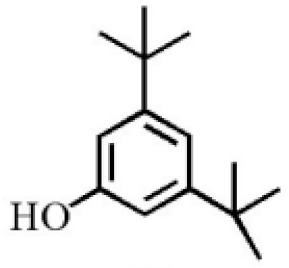	
4-Methoxyphenylacetone	0.95	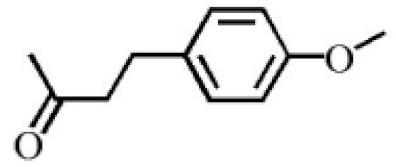	

**Table 3:** Chemical structure of 2-(2-phenylethyl) chromones in agarwood.

Name	Chemical Structure	Reference
2-[2-(4-Hydroxyphenyl)ethyl]chromone	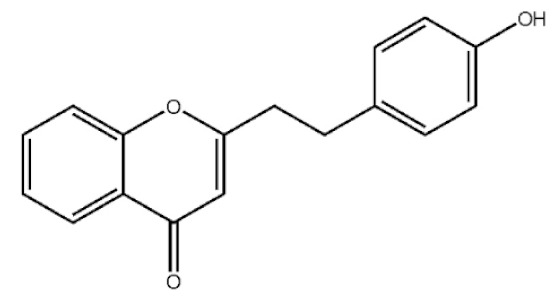	[33]
5,6,7,8,-Tetrahydroxy-5,6,7,8-tetrahydro-2-[2-(4-methoxyphenyl)ethy]-chromone	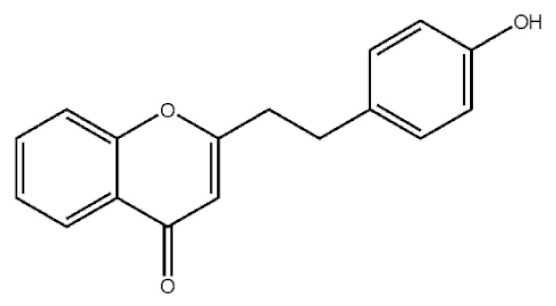	
*Rel-*(1*AR*,2*R*,3*R*,7*bS*)-1a,2,3,7b-Tetrahydro-2,3-hidydroxy-5[2-(4-methoxyphenyl)ethy]-7*H*-oxireno[f][1]benzophran-7-one	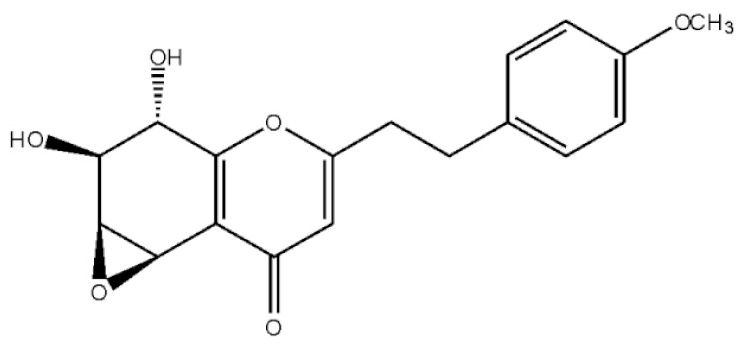	
Oxidoagarchromones A	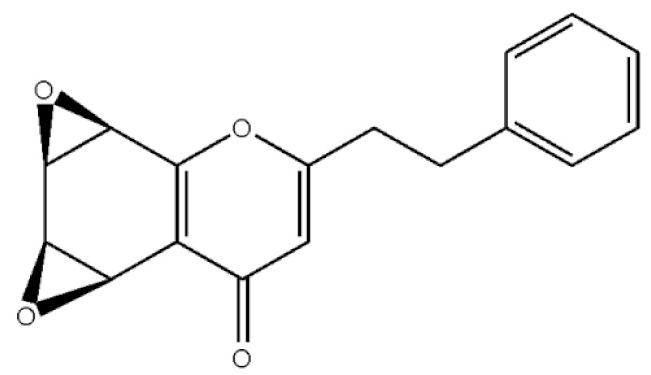	
6,8′-Dihydroxy-2-2′-bis(2-phenylethyl)-4*H*,4′*H*-5,5′-bichromone-4,4′-dione	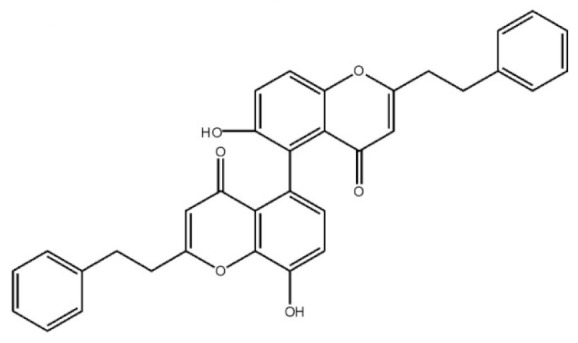	
Agarotetrol	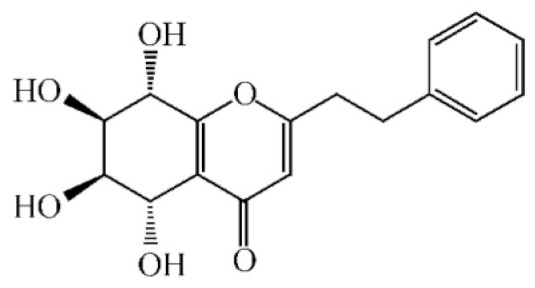	[34,35]
Isoagarotetrol	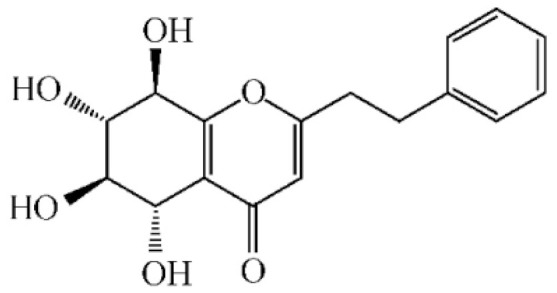	
2-(2-phenylethyl) chromone	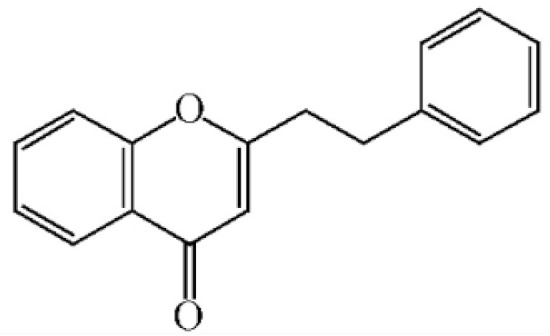	
2-[2-(4-methoxyphenyl) ethyl] chromone	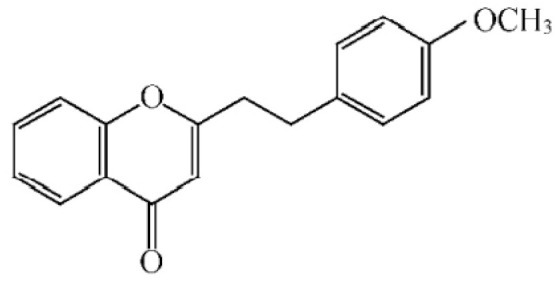	

**Table 4:** Chemical structure of terpenoids from agarwood.

Name	Chemical Structure	Reference
7α,15-Dihydroxydehydroabietic acid	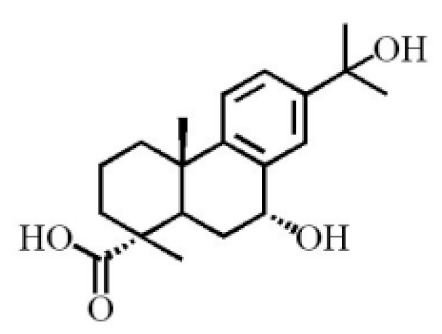	[46]
Methyl 7-oxodehydroabietate	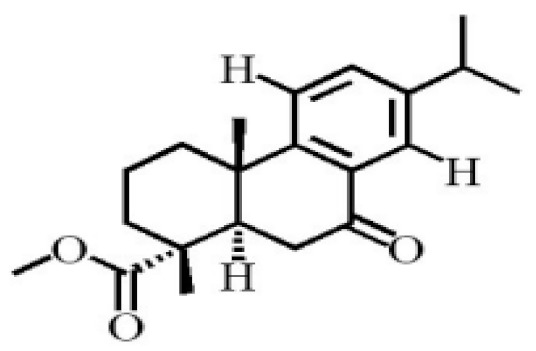	
7α-hydroxypodocarpen-8(14)-en-13-on-18-oic acid	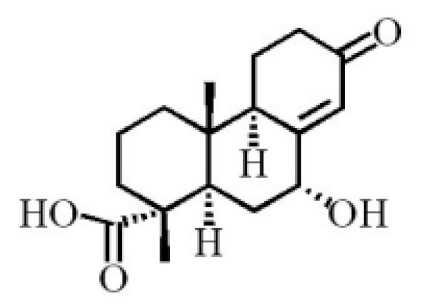	
18-norpimara-8(14),15-dien-4αα-ol	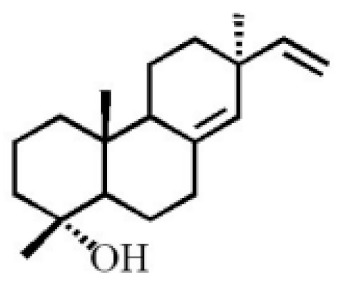	
18-norpimara-8(14), and 15-dien-4α-ol	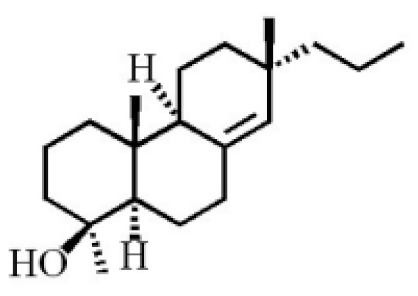	
7α, 12α, 13α-trihydroxyabiet-8(14)-en-18-oic acid acetonide	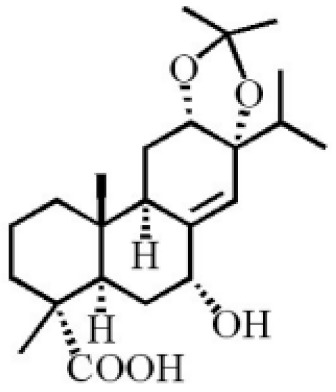	[47]
6α, 13α, 14α-trihydroxyabiet-7-en-18-oic acid	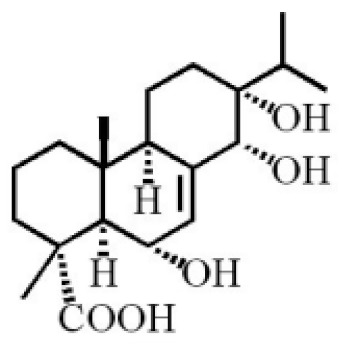	
13α, 14α, 15-trihydroxy-7-oxoabiet-8-en-18-oic acid	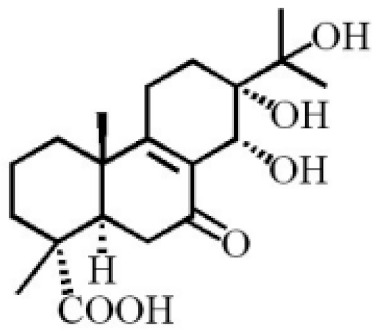	
13β, 14β-epoxyabiet-7-en-18, 6α-olide	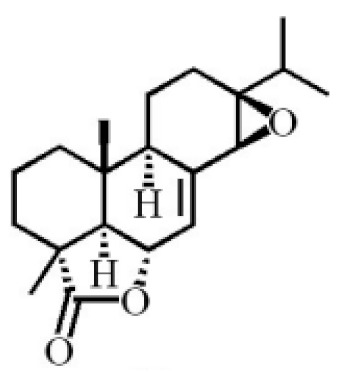	
7α, 12β, 13β-trihydroxyabiet-8(14)-en-18-oic acid	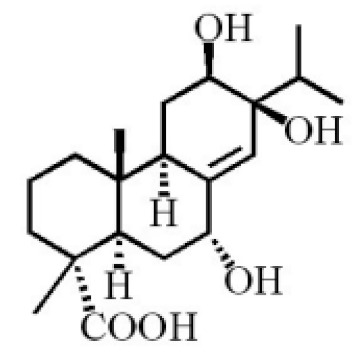	
7α-hydroxyabieta-15-methoxy-8,11,13-trien18-oic acid	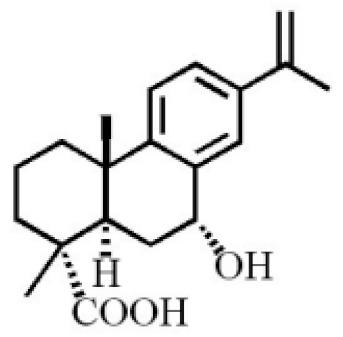	
7α-hydroxyabieta-15-methoxy-8,11,13-trien18-oic acid	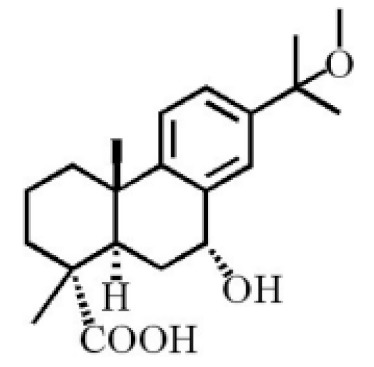	
12α-ethoxyabieta-7,13-dien-18-oic acid	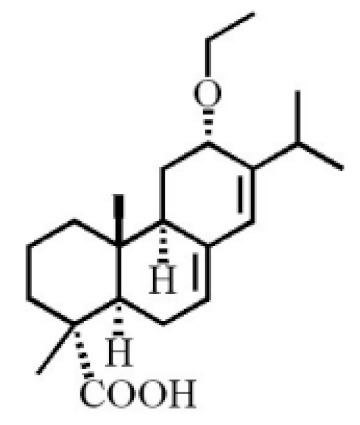	
7,13-dioxopodocarpan-18-oic acid	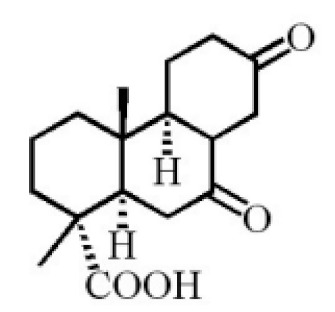	
α-Agarofuran	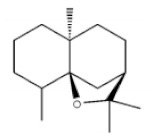	[36]
(5*S*,7*S*,10*S*)-(-)Selina-3,11-dien-9-one	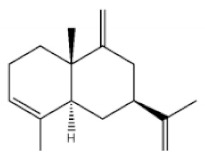	
(+)-(4*S*,5*R*)-Dihydrokaranone	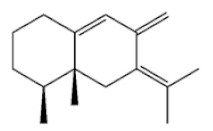	
α-Guaiene	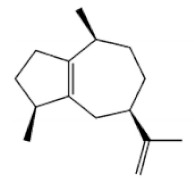	
Agarospirol	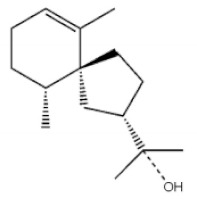	
8-β-*H*-Dihydrogmelofuran	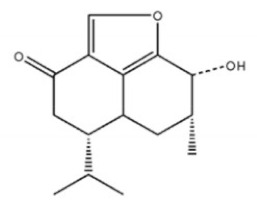	
(-)-bornyl ferulate	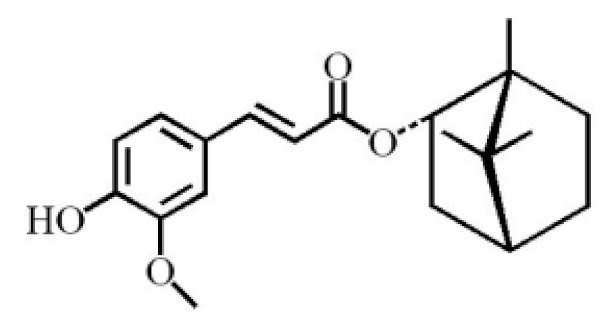	[37]

**Table 5:** A summary of pharmacological effects of agarwood.

Pharmacological Action	Active Substance	Action Mechanism
Antibacterial and bacteriostatic	Sesquiterpenes	Its antibacterial mechanism can induce cell apoptosis through the process of nuclear condensation and cleavage.
Anti-tumor	Sesquiterpene, chromone, and triterpene	Its mechanism may be related to the induction of apoptosis through nuclear condensation and breakage, including the destruction of mitochondrial membrane potential.
Sedation, analgesia, and anti-inflammation	Sesquiterpene and chromone	The mechanism of analgesia and sedation may be related to the regulation of gene expression of GABAA receptor, enhancement of GABAA receptor function, and promotion of Cl^−1^ influx, and the anti-inflammatory mechanism may be related to the inhibition of granulocyte respiratory burst, inhibition of pro-inflammatory cytokines (IL-1 β, IL-6, and TNF- α), and decrease in lipid peroxidation (MDA).
Relieving cough and relieving asthma	Terpenoid/chromone	It is speculated that the mechanism by which agarwood relieves asthma may be related to anti-inflammation, anti-apoptosis, improvement of pathological changes of lung and an intestinal tract, abnormal function, and balance of immunity.
Antidepressant	Diterpene	The mechanism may be related to the inhibition of the corticotropin-releasing factor (CRF) gene expression and the hyperactivity of the hypothalamus–pituitary–adrenal (HPA) axis, as well as the inhibition of corticotropin receptor gene transcription and protein expression in the cerebral cortex and hippocampus.
Anti-oxidation and anti-aging	Flavonoids and sesquiterpenes	The mechanism may be related to the regulation of reactive oxygen clusters and proinflammatory cytokines by microglia and the release of stress hormones.
Cardiovascular system	Chromone	The mechanism may inhibit cardiomyocyte apoptosis after ischemia/reperfusion by regulating B lymphocyte tumor-2 gene (Bcl-2) and to down-regulating rabbit anti-human monoclonal antibody (Bax).

**Table 6:** Quality grading system of agarwood.

Morphological Feature	Grade		
	A	B	C
Color	Dark brown or khaki with a small number of white spots	Yellowish or reddish-brown with more white spots	Khaki or yellowish-brown with a large number of white spots
Density of resin	Very dense and compact, sink in water when soaked	Dense and less compact, half-sinkage in water	Light and not dense, full-floating on water
Weight	Hard texture, brittle, and not hollowed	The texture is a little hard, a little brittle, and slightly hollow	Loose texture, not brittle, and hollow
Aroma	Strong odor, feel sweet and cool.	Less potent odor, feel sweet and slightly spicy	The aroma is light, feel slightly sweet and salty
Sense of oiliness	Have a strong sense of oiliness	Have a strong sense of oiliness	The sense of oiliness is weak

## Data Availability

The data presented in this study are available on request from the corresponding author.

## Sample Availability

Samples of the compounds are not available from the authors.
